# Research on the estimation method of crop net primary productivity based on improved CASA model

**DOI:** 10.3389/fpls.2025.1659047

**Published:** 2025-11-03

**Authors:** Wanning Li, Zhuo Wang, Chunling Chen, Ying Yin, Yuanji Cai, Hao Han, Minghuan Liu, Ziyi Feng

**Affiliations:** ^1^ College of Information and Electrical Engineering, Shenyang Agricultural University, Shenyang, China; ^2^ Henan Provincial Key Lab of Hydrosphere and Watershed Water Security, North China University of Water Resources and Electric Power, Zhengzhou, China

**Keywords:** CASA model, crop monitoring, net primary productivity (NPP), Fraction of Photosynthetically Active Radiation (FRAR), vegetation index

## Abstract

Net Primary Productivity (NPP) is a vital indicator for evaluating the carbon source and sink capacities of ecosystems, significantly influencing assessments of agricultural productivity and carbon cycle studies. Accurately estimating NPP in the agricultural sector, however, remains challenging. This research addresses the challenge by refining the estimation of the Fraction of Photosynthetically Active Radiation (FPAR) within the CASA model, introducing a novel methodology that significantly improves the accuracy of NPP estimation and, when applied to remote sensing imagery covering a broad region, demonstrates strong potential for large-scale crop NPP monitoring. We employed high-resolution Sentinel-2 satellite imagery and the Recursive Feature Elimination algorithm to extract FPAR-related features from 15 vegetation indices. The FPAR was subsequently estimated using a Convolutional Neural Network, leading to a dramatic decrease in the Root Mean Square Error (RMSE) from 0.2040 to 0.0020. The prediction errors for the improved model ranged from 0.0001 to 0.0092, with a mean absolute error (MAE) below 0.01. These values reflect the distribution of absolute residuals and indicate a substantial enhancement in accuracy over traditional methods. This improved FPAR estimation method was subsequently integrated into the CASA model. Compared to field-measured NPP data, the optimized model reduced the Mean Absolute Percentage Error (MAPE) from 28.92% to 20.31%. The MAPE values across the test samples ranged between 15% and 25%, indicating a significant improvement in model reliability. The optimized CASA model performs well in estimating net primary productivity (NPP) of crops, providing strong support for agricultural decision-making and future research on large-scale productivity and carbon cycling.

## 1 Introduction

Vegetation productivity plays a vital role in supporting human life by providing food, raw materials, and energy, with edible crops serving as the primary source of nutrition (Running, 2012). Net Primary Productivity (NPP) (Zhao and Running, 2010; Tian et al., 2016) is a key metric of vegetation productivity, reflecting the production potential of plant communities within their natural environments, and serving as a crucial indicator for assessing carbon sources and sinks within ecosystems (Zeng et al., 2023). Accurate monitoring and estimation of NPP of crop vegetation is essential for optimizing crop cropping structure and improving agricultural yields (Myneni et al., 2001; Heinsch et al., 2006).Traditionally, NPP is measured by *in situ* observation, which is accurate at a local scale but is operationally cumbersome, resource consuming, and not applicable to large-scale monitoring (Veroustraete et al., 2002; Wang et al., 2010; Yang et al., 2017). Advances in remote sensing and data processing have provided three main approaches for estimating NPP: statistical models, process-based models, and light use efficiency (LUE) models (Feng et al., 2007). Statistical models extrapolate measured data based on climate correlations, but lack stability and scalability (Net primary productivity in the terrestrial biosphere: The application of a global model, 1994). Process-based models incorporate plant physiological and ecological mechanisms and can provide reliable estimates, but require a large number of parameters and are therefore limited in spatial applicability (Running et al., 2004). The LUE model, which estimates NPP based on the absorption of solar radiation by vegetation as well as moderating factors, combines simplicity, adaptability, and applicability, and is well suited for long-term and large-scale remote sensing applications (Field et al., 1995).

The Carnegie-Ames-Stanford Approach (CASA) model is a widely applied light-use efficiency model for estimating vegetation NPP (Potter et al., 1993). The model suggests that NPP is influenced by the quantity of photosynthetically active radiation (PAR) taken in by vegetation and the effectiveness with which plants transform this absorbed radiation into biomass. A key parameter within the CASA model is the Fraction of Absorbed Photosynthetically Active Radiation (FPAR) absorbed by vegetation (Cheng et al., 2014). When incident PAR (400-700nm) reaches the top of the vegetation canopy, a portion is reflected, a portion is absorbed by the vegetation, and another portion is transmitted to the ground. Only the PAR absorbed by the vegetation canopy contributes to biomass accumulation. FPAR represents the ratio of PAR absorbed by the canopy to the incident PAR (Gitelson et al., 2015). Accurate estimation of FPAR is crucial for assessing NPP, as it directly affects the amount of energy available for photosynthesis, thereby influencing the overall productivity of the ecosystem.

Traditional methods estimate FPAR by leveraging the statistical correlation between FPAR and spectral vegetation indices, calibrating these indices with field-measured FPAR data. However, these methods have limited spatiotemporal scalability, and their accuracy depends on data from specific times and locations. Another approach involves FPAR retrieval based on canopy radiative transfer models, but the computational demand is significant, limiting its application on a large scale (Peng et al., 2018; Liang et al., 2023). Wang et al. developed a 10-meter FPAR algorithm for Sentinel-2 data by analyzing the correlation between MODIS FPAR and surface reflectance from Sentinel-2 (Wang et al., 2022). Fang et al. improved FPAR retrieval accuracy by analyzing differences between red-edge and non-red-edge indices. Their results demonstrated that the red-edge normalized difference vegetation index and red-edge simple ratio vegetation index achieved higher FPAR retrieval accuracy than the original CASA model (Fang et al., 2021). Similarly, Gao et al. proposed a deep learning-based algorithm for retrieving FPAR from MODIS visible-band surface reflectance. The model, trained on simulated data, enhances the inversion process while maintaining accuracy. Validation against MODIS FPAR products and ground-based measurements demonstrates its effectiveness, particularly in regions lacking vegetation classification (Gao et al., 2020a). Within the CASA model, FPAR estimation commonly relies on statistical relationships with vegetation indices, such as NDVI and RVI (Yang, 2022). However, these simple regression models fail to fully exploit the rich information available in satellite data. Current FPAR inversion methods face several challenges, such as regional specificity and variability across different vegetation types, limiting their general applicability. To address these limitations, this study trains convolutional neural networks (CNNs) using a variety of vegetation indices and FPAR data. The inclusion of multiple vegetation indices, which encompass diverse information such as soil background and moisture conditions, enhances the model’s adaptability. CNNs, as a deep learning framework, excel at automatically learning complex features from high-dimensional data, making them well-suited for estimating FPAR from vegetation indices derived from remote sensing imagery (Wang et al., 2023). Compared to traditional methods, CNNs can more effectively utilize the wealth of information in satellite data, improving both the accuracy and efficiency of FPAR estimation (Gao et al., 2020b; Alzubaidi et al., 2021; Wang et al., 2023).

This study seeks to accomplish the following primary objectives: (1) To develop a deep learning model that enhances the accuracy of FPAR estimation using high-resolution satellite imagery and vegetation indices from different growth stages; (2) To integrate the deep learning framework with the CASA model for evaluating NPP estimation accuracy and validating the enhanced model’s reliability; (3) To analyze the characteristics and driving factors of NPP results. Through these efforts, we seek to improve the precision and applicability of NPP estimation, advance large-scale monitoring of crop NPP, and provide a scientific basis for agricultural management and policy-making.

## 2 Materials and methods

**Figure 1:**
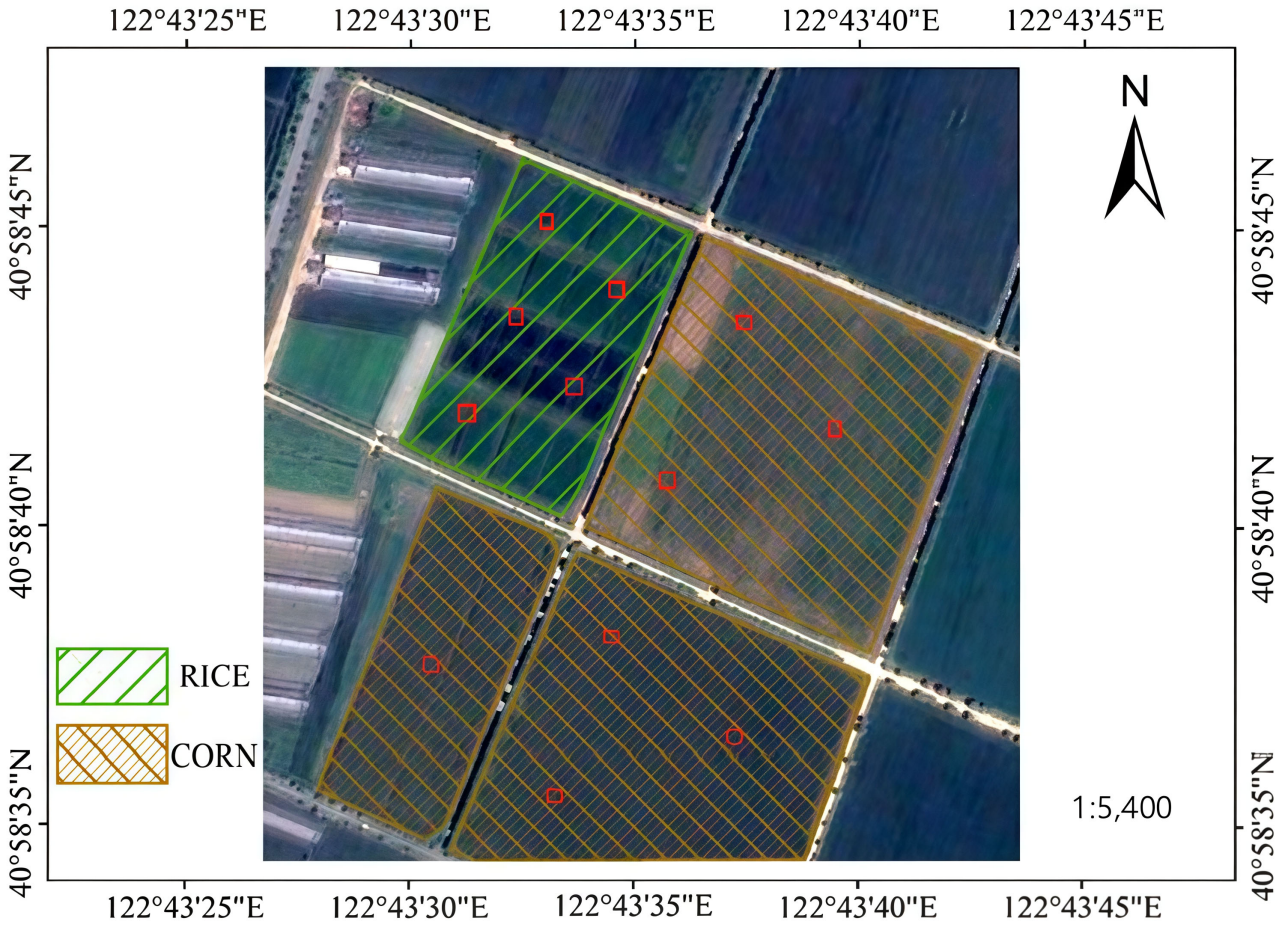
Overview of the Study Area.(The cropping structure is shown, where green diagonal hatching represents rice fields and brown diagonal hatching represents corn fields).

Haicheng City in Liaoning Province, located at 122°39′18″E and 40°58′58″N, experiences a warm temperate climate with an annual average temperature of 9.3 °C and total precipitation of 710.2 mm per year (Li et al., 2020). The study area is located at the Shenyang Agricultural University experimental base in Gengzhuang Town, Haicheng City. This region is characterized by high soil organic matter content, with surface soil pH values ranging from 6.5 to 7.2 (Lal, 2004), indicating moderate soil fertility and suitability for plant growth. The primary cereal crops in the study area are corn and rice, with the cropping structure shown in **Figure 1**. The local cropping system consists of a single annual crop cycle, where the growing season lasts from April to October.

In 2022, we conducted field measurements of NPP in the study area. The measurements were conducted at the Shenyang Agricultural University experimental base in Haicheng City, where we selected five rice fields and seven corn fields, with specific latitude and longitude coordinates provided in **Table 1**. Before crop harvest, plant samples were collected using sampling plots measuring 1 meter by 1 meter. Within each plot, plants were cut at ground level and sectioned into approximately 25 cm segments. These samples were first heated in an oven at 105 °C for 30 minutes to halt biological activity. The oven temperature was then reduced to 65 °C and the samples were dried to a constant weight. We then measured the dry weight of the plants in each plot using a high-precision balance and ground the samples into a fine powder for carbon content analysis. By multiplying the average carbon content of the plants in each plot by their dry weight, we calculated the organic matter content associated with plant growth and reproduction, which represents the NPP (Ni, 2004). The average NPP value for each experimental field was obtained by averaging the NPP values from all plots within that field. Ultimately, we calculated the average NPP values for 12 experimental fields, offering data support for assessing the performance of the improved CASA model.

**Table 1:** Latitude and longitude coordinates of corn and rice sampling points.

ID	Crop type	Longitude (°E)	Latitude (°N)
1	corn	122.726279	40.973931
2	corn	122.726421	40.969247
3	corn	122.726750	40.968754
4	corn	122.727033	40.967275
5	corn	122.727174	40.969740
6	corn	122.726892	40.970726
7	corn	122.726609	40.966289
8	rice	122.725007	40.974670
9	rice	122.725290	40.973684
10	rice	122.725479	40.975656
11	rice	122.725667	40.972698
12	rice	122.725102	40.972205

Meteorological data for this study were sourced from the Xiaomaiya Agricultural Meteorological Big Data System platform (https://wheata.cn/). We downloaded monthly records of temperature, precipitation and solar radiation for the study area for the year 2022. Temperature was recorded in degrees Celsius (°C), precipitation was measured in millimeters (mm), and solar radiation data were initially provided in joules per square meter per day (J/m²/day). For ease of analysis, the solar radiation data were converted to megajoules per square meter per month (MJ/m²/month). The organized data for each month, including total monthly radiation, average monthly temperature, and monthly precipitation, along with the corresponding latitude and longitude information, were imported into the ArcGIS platform in tabular format. Kriging interpolation, a geostatistical method that accounts for spatial correlation, was employed to process the data, generating more scientifically accurate and realistic interpolation results (Li et al., 2023). This approach generated monthly aggregates of solar radiation, average temperatures, and precipitation data for each pixel within the study area.

This study employs Sentinel-2 satellite imagery as the main source of remote sensing data (Drusch et al., 2012). Sentinel-2 provides high-resolution multispectral images across 13 spectral bands with spatial resolutions of 10 meters, 20 meters, and 60 meters (Immitzer et al., 2012). Sentinel-2 imagery can be accessed via the Copernicus Open Access Hub (https://browser.dataspace.copernicus.eu). The mission comprises two satellites, Sentinel-2A and Sentinel-2B, which together ensure a 10-day revisit period. When operating in tandem, the revisit period is reduced to 5 days, providing enhanced temporal resolution. Additionally, Sentinel-2 includes three bands in the red-edge spectrum, which enhances its utility for agricultural monitoring (d’Andrimont et al., 2020). Due to its excellent spatiotemporal resolution and extensive spectral coverage, Sentinel-2 data are used as the primary input for the CASA model in this study, facilitating high-precision crop monitoring and comprehensive analysis of NPP (Wulder et al., 2012).

**Figure 2:**
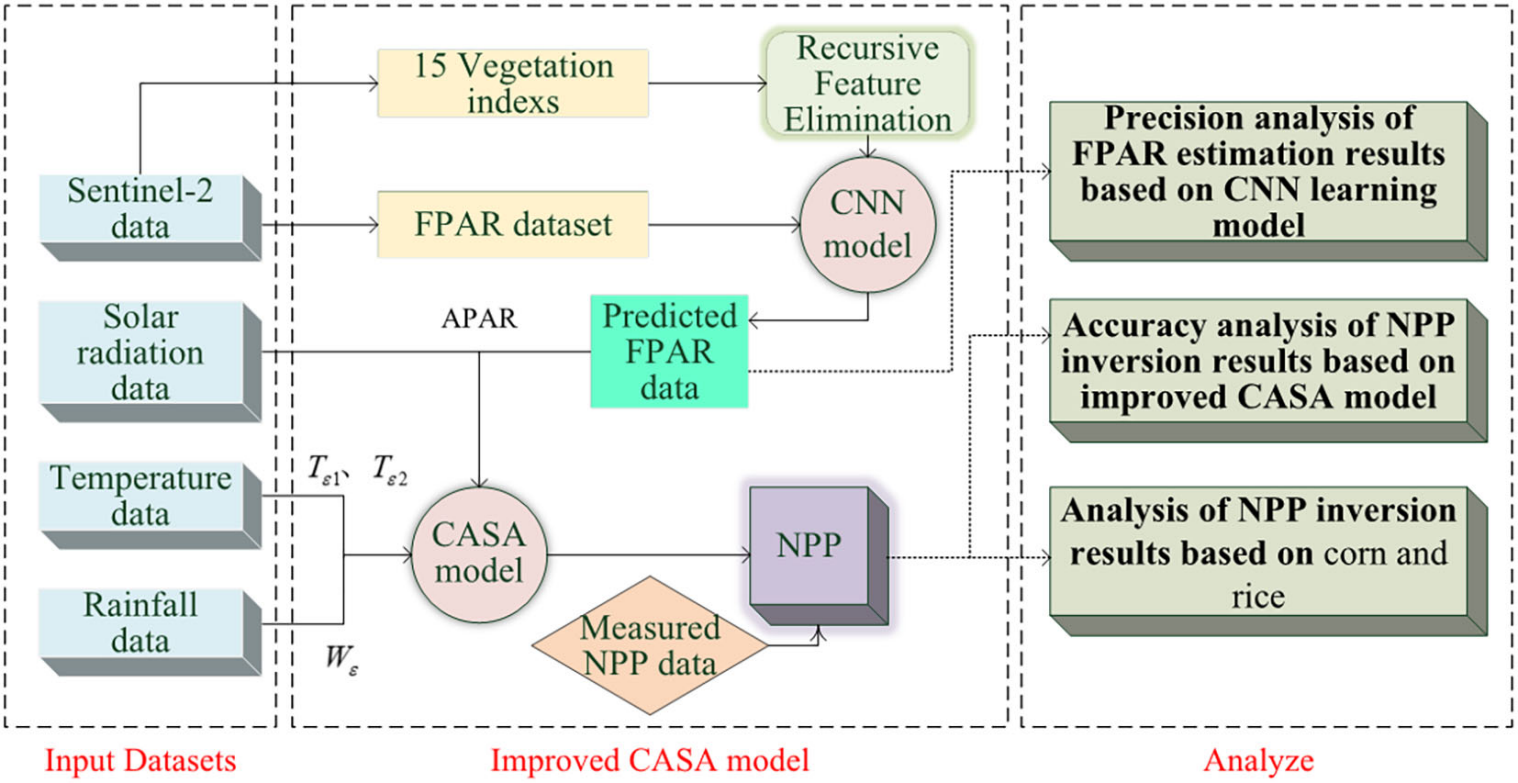
Workflow of this study’s methodology.

This study aims to enhance the accuracy of FPAR and NPP estimations by refining the FPAR parameter estimation method within the CASA model. The technical workflow is illustrated in **Figure 2**. To achieve this, we first utilized Sentinel-2 satellite imagery, along with data on solar radiation, temperature, and precipitation. We then compiled a dataset consisting of 15 vegetation indices related to crops and their corresponding FPAR values for training a CNN regression model. A feature selection method based on recursive feature elimination was used to identify the optimal set of indices, which was then applied to train the CNN model. The trained model was subsequently applied to estimate FPAR. We integrated the model-predicted FPAR results with solar radiation, precipitation, and temperature data, which were then input into the CASA model to obtain optimized NPP results. Finally, we validated the CNN model’s FPAR estimates and compared the improved CASA model’s NPP results with field-measured NPP data. Further analysis was conducted on the NPP estimation results for rice and corn.

To identify vegetation indices relevant to crop FPAR estimation, this study selected 15 variables for FPAR inversion. These include the Atmospherically Resistant Vegetation Index (ARVI), Difference Vegetation Index (DVI), Enhanced Vegetation Index (EVI), Global Environment Monitoring Index (GEMI), Green Normalized Difference Vegetation Index (GNDVI), Modified Soil Adjusted Vegetation Index (MSAVI), Normalized Difference Index (NDI45), Normalized Difference Vegetation Index (NDVI), Perpendicular Vegetation Index (PVI), Ratio Vegetation Index (RVI), Red-Edge Inflection Point Index (REIP), Soil Adjusted Vegetation Index (SAVI), Transformed Normalized Difference Vegetation Index (TNDVI), Transformed Soil Adjusted Vegetation Index (TSAVI), and Weighted Difference Vegetation Index (WDVI). Among them, EVI and GEMI are notable for their proven significant advantages in reducing background effects and improving the accuracy of FPAR estimates. The EVI was developed by Dai Liang Peng to improve upon traditional vegetation indices like NDVI by minimizing atmospheric influences and surface reflectance variations. This index has demonstrated enhanced sensitivity in capturing vegetation dynamics, leading to more accurate estimates of the FPAR. Additionally, the GEMI, based on research by Leolini, demonstrated optimal performance in estimating FPAR for olive trees during arid periods with low grass cover (Leolini et al., 2022).

To obtain the 15 vegetation indices, this study first resampled Sentinel-2 Level-2A data from the Copernicus Open Access Hub (https://dataspace.copernicus.eu/), covering the crop growing season from April to October 2022 with minimal cloud cover. These data were subsequently resampled on the SNAP platform by bilinear sexual interpolation to obtain a uniform 10 m spatial resolution. This resampling process standardized the spatial resolution of the various bands to 10 meters. Next, the Band Math tool in ENVI was employed to compute the 15 commonly used vegetation indices, with their calculation formulas provided in **Table 2** (Srivastava et al., 2014; Sonobe et al., 2018; Kumar et al., 2022).

**Table 2:** Formulas for standard vegetation Indices (where B2, B4, B5, B7, B8, and B8A refer to Sentinel bands; a denotes the soil line intercept; b indicates the soil line slope; X is the factor used to reduce soil noise; and L is the calibration factor).

Index name	Definition of indices
ARVI (Huete et al., 1997)	$\frac{B8-B4-2\times (B4-B2)}{B8+B4-2\times (B4-B2)}$
DVI (Gunathilaka, 2021)	B8−B4
EVI (Huete et al., 1997)	$2.5\times \frac{B8-B4}{B8+6\times B4-7.5\times B2+1}$
GEMI (Pinty and Verstraete, 1992)	$\left(n\times (1-0.25\times n)-\frac{B4-0.125}{1-B4}\right)$ $n=\frac{2\times (B8A^{2}-B4^{2})+1.5\times B8A+0.5\times B4}{B8A+B4+0.5}$
GNDVI (Lobell and Asner, 2003)	$\frac{B7-B3}{B7+B3}$
MSAVI (Chen et al., 2010)	$\frac{2\times B8+1-\sqrt{{\left(2\times B8+1\right)}^{2}-8\times (B8-B4)}}{2}$
NDI45 (Delegido et al., 2011)	$\frac{B5-B4}{B5+B4}$
NDVI (Tucker, 1979)	$\frac{B8-B4}{B8+B4}$
PVI (Richardson and Wiegand, 1977)	$\frac{B8-a\times B4-b}{\sqrt{{\text{a}}^{2}+1}}$
RVI (Gupta, 1993)	$\frac{B8}{B4}$
REIP (Delegido et al., 2011)	$705+35\times (\frac{B4+B7}{2}-\frac{B5}{B6-B5})$
SAVI (Baret and Guyot, 1991)	$(1+L)\times \frac{B8-B4}{B8+B4+L}$
TNDVI (Baret et al., 1989)	$\sqrt{\frac{B8-B4}{B8+B4}+0.5}$
TSAVI (Baret et al., 1989)	$\frac{s\times (B8-s\times B4-a)}{a\times B8+B4-a\times s+X\times (1+s^{2})}$
WDVI (Clevers, 1989)	B8−a×B4

Meanwhile, a biophysical processor tool was used in the Sentinel Application Platform (SNAP) to generate FPAR data at 10 m resolution. The processor uses a neural network trained on a synthetic database generated by the radiative transfer model to estimate three key biophysical variables: leaf area index (LAI), photosynthetically active radiation absorption ratio (FPAR), and vegetation cover ratio. The database accurately modelled canopy reflectance within the Sentinel-2 spectral band and was trained separately for the S2A and S2B sensors to account for their unique spectral response characteristics. According to the SNAP documentation, the FPAR neural network achieved high accuracy, with R-squared (R^2^) values of 0.92 and 0.91 for S2A and S2B, respectively, and an Root Mean Square Error (RMSE) value of 0.072 for each (Weiss and Jay). These results indicate that the processor is capable of generating reliable and high-quality FPAR estimates. In this study, the FPAR derived from SNAP is used as the reference data for training CNN model.

In order to build a robust FPAR inversion model, we calculated 15 vegetation indices from the resampled Sentinel-2 data. To reduce redundancy and improve model performance, we used the recursive feature elimination (RFE) algorithm to rank and select the most relevant indices (Jeon and Oh, 2020). RFE iteratively removes features that contribute the least to model performance and retains only those features that significantly improve prediction accuracy. The formulas for these indices are listed in **Table 2**.

To estimate FPAR, three machine learning models (CNN, GBDT, and XGBoost) were constructed. The performance of each model was evaluated using four metrics: R², MSE, EVS, and MAE.

Compared to tree-based models, which are good at dealing with structured data and feature interactions, convolutional neural networks show superior ability in capturing spatial-style and non-linear relationships, making them more suitable for processing high-dimensional remote sensing data (Sagi and Rokach, 2021).

**Figure 3:**
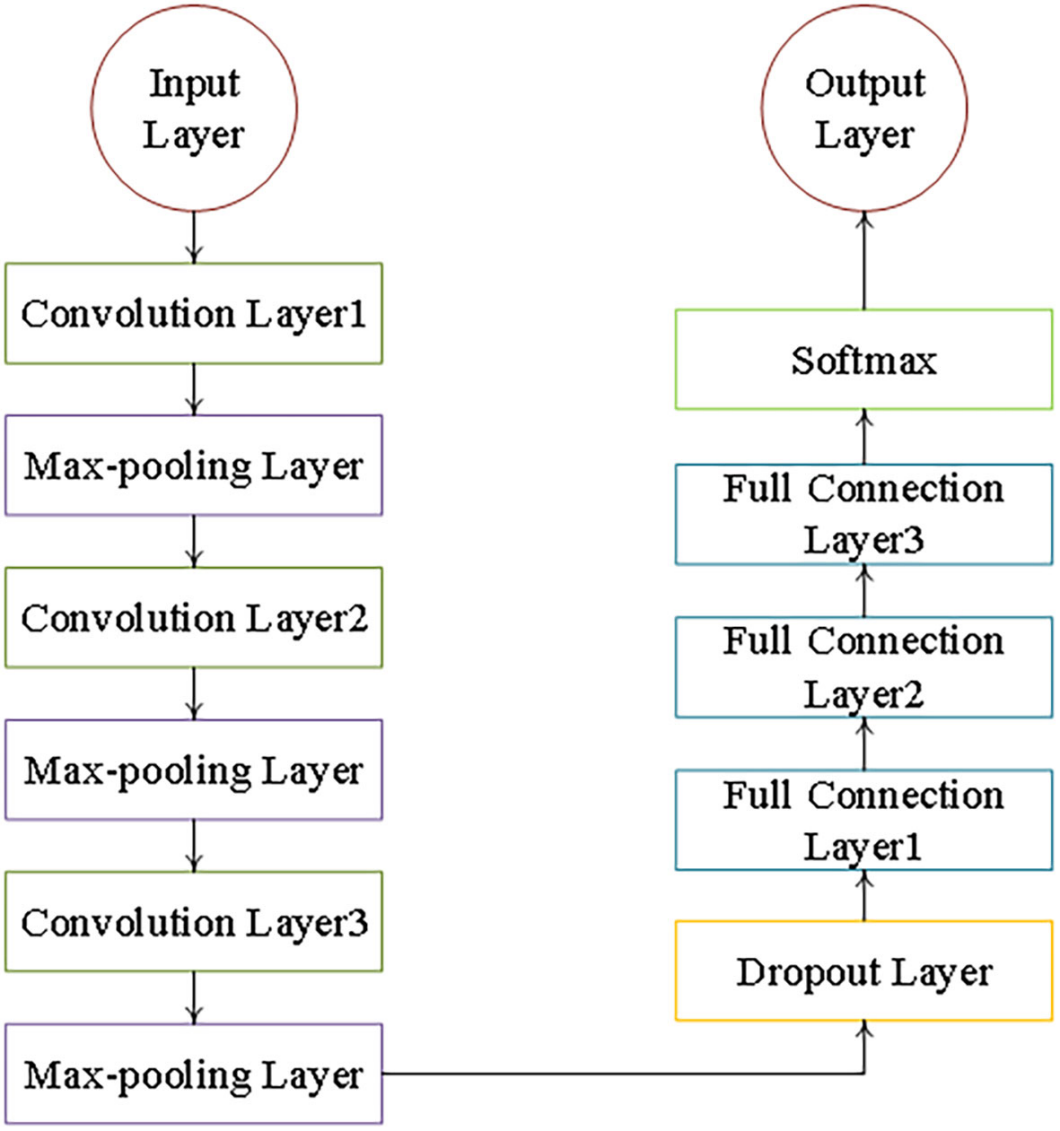
CNN model architecture.

The CNN architecture shown in **Figure 3** contains an input layer, three convolutional layers, three pooling layers, a Dropout layer, three fully connected layers, and finally a softmax activation function. The data fed into the CNN consisted of 2,756 vegetation index samples selected by the RFE, which had been normalized according to the FPAR values (Alzubaidi et al., 2021).The dataset was divided into training and test sets in a ratio of 7:3 and was subjected to 10 training cycles to ensure model convergence.

In order to improve the accuracy and efficiency of FPAR estimation, a convolutional neural network (CNN) model was constructed and optimized with a combination of grid search and Bayesian optimization techniques. The optimized CNN architecture consists of three convolutional layers, each with 16, 32 and 64 filters respectively, each with a kernel size of 2. Training was performed using an Adam optimizer with a learning rate of 0.001. The mean squared error (MSE) was used as the loss function. To reduce overfitting, a Dropout layer (ratio = 0.3) was added before the fully connected layer, and 30% of the neurons were randomly deactivated during training. **Table 3** shows the detailed configuration of the CNN model and hyperparameters. The final model achieves high predictive performance on the validation and test sets while maintaining computational efficiency.

**Table 3:** Configuration and optimized parameters of the CNN model.

Parameter	Value
Input Features	15 Vegetation Indices
Conv Layers & Filters	3 layers (16, 32, 64 filters)
Kernel Size	2
Dropout Rate	0.3
Optimizer	Adam (lr = 0.001)
Loss Function	MSE
Evaluation Metrics	R², MSE, MAE, EVS
Optimization Method	Grid Search + Bayesian Tuning

The CASA model estimates NPP based on two key variables: absorbed photosynthetically active radiation (APAR) and actual light use efficiency (LUE) (Equation 1) (Yu et al., 2009). In the modified CASA model, FPAR is predicted by the method described in Section 2.3.2, whereas the original CASA model derives FPAR based on the empirical relationship between NDVI and RVI vegetation indices. APAR represents the absorbed photosynthetically active radiation per unit area, and is determined by surface solar radiation and vegetation FPAR (Equation 2).


(1) $$\begin{array}{c} NPP\left(x,t\right)=APAR\left(x,t\right)\times \epsilon \left(x,t\right) \end{array}$$

(2) $$\begin{array}{c} APAR\left(x,t\right)=SOL\left(x,t\right)\times FPAR\left(x,t\right)\times 0.5 \end{array}$$

Changes in actual LUE were mainly influenced by temperature and water stress Equation 3). The temperature stress factors Te1 and Te2 correspond to the effects of low and high temperatures, respectively, while Werep represents the effect of water availability. The maximum light use efficiency (Emax) reflects the optimum growing conditions and varies with vegetation type; in this study, it was set at 0.389 g C/MJ for the crop in the study area (Yu et al., 2009).


(3) $$\begin{array}{c} \epsilon \left(x,t\right)=T_{\epsilon 1}\left(x,t\right)\times T_{\epsilon 2}\left(x,t\right)\times W_{\epsilon }\left(X,T\right)\times \epsilon_{max} \end{array}$$

To ensure that the NPP estimates reflect the crop growing season, data from April to October were used and vegetation data outside this period were excluded. As a result, the CASA model combines factors such as light, temperature and humidity to simulate vegetation productivity. The proposed improvements improve the accuracy of the model under variable environmental conditions and support ecosystem productivity studies by combining remotely sensed data and ground-based observations.

Model performance was assessed using five metrics: R^2^ (Equation 4), Mean Squared Error (MSE, Equation 6) Explained Variance Score (EVS, Equation 5), Mean Absolute Error (MAE, Equation 7), and Mean Absolute Percentage Error (MAPE, Equation 8). R^2^ (Equation 4) measures the proportion of the variance in the dependent variable that is explained by the independent variable, and ranges from 0 to 1, with higher values indicating a better fit. Higher values indicate a better fit. EVS (Equation 5) assesses the consistency of the predictions, also ranging from 0 to 1 Higher values indicate greater explanatory power. These metrics complement each other, with R^2^ (Equation 4) reflecting the total explained variability and EVS (Equation 5) reflecting the consistency of the predictions with the observations.


(4) $$\begin{array}{c} R^{2}=1-\frac{\sum_{i}{\left({\hat{y}}_{i}-y_{i}\right)}^{2}}{\sum_{i}{\left(\bar{y_{i}}-y_{i}\right)}^{2}} \end{array}$$

(5) $$\begin{array}{c} EVS=1-\frac{Var\left(y_{i}-{\hat{y}}_{i}\right)}{Var\left(y_{i}\right)} \end{array}$$

MSE (Equation 6) and MAE (Equation 7) quantify prediction error in different ways. MSE (Equation 6) calculates the mean of the squared difference between the predicted and observed values, and is therefore more sensitive to large deviations, and is expressed in squared units. In contrast, MAE (Equation 7) calculates the mean of the absolute errors, maintaining the same units as the observations and thus providing a more intuitive interpretation. MAPE (Equation 8) expresses the prediction errors as a percentage, allowing for comparisons that are not scale-dependent. A value of 0%for MAPE (Equation 8) indicates a perfect prediction, whereas a value greater than 100 per cent suggests that the prediction is performing poorly. Together, these metrics provide a comprehensive assessment of the predictive accuracy of the model.


(6) $$\begin{array}{c} MSE=\frac{1}{n}\sum_{i}{\left(y_{i}-{\hat{y}}_{i}\right)}^{2} \end{array}$$

(7) $$\begin{array}{c} MAE=\frac{1}{n}\sum_{i}\left|y_{i}-{\hat{y}}_{i}\right| \end{array}$$

(8) $$\begin{array}{c} MAPE=\frac{1}{n}\sum_{i}\left|\frac{y_{i}-{\hat{y}}_{i}}{y_{i}}\right| \end{array}$$

## 3 Results

### 3.1 FPAR estimation results based on the CNN learning model

This study performed a correlation analysis between vegetation indices and FPAR for different months, with the results presented in **Table 4**. The results of the study showed that the highest and most stable correlation was found between SAVI and FPAR, which was old enough to adjust soil brightness and reduce background effects (McDonald et al., 1998; Zhu et al., 2014). However, further feature selection analyses showed that metrics such as GEMI, NDI45 and RVI contributed more significantly to model performance, highlighting that correlation does not necessarily equate to the greatest predictive importance during training.

**Table 4:** Correlation analysis between FPAR and vegetation indices for different months.

Vegetation indices	May	June	July	August	September
ARVI	0.97	0.93	0.96	0.79	0.97
DVI	0.94	0.95	0.97	0.89	0.93
EVI	0.97	0.98	0.09	0.63	0.94
GEMI	0.91	0.91	0.65	0.84	0.89
GNDVI	0.81	0.92	0.96	0.86	0.80
MSAVI	0.97	0.98	0.98	0.98	0.95
NDI45	0.79	0.65	0.80	0.79	0.80
NDVI	0.99	0.98	0.97	0.86	0.97
PVI	0.98	0.96	0.73	0.92	0.94
REIP	0.01	0.41	-0.01	0.34	0.32
RVI	0.98	0.96	0.94	0.87	0.61
SAVI	0.97	0.99	0.98	0.98	0.97
TNDVI	0.98	0.98	0.96	0.86	0.97
TSAVI	0.99	1	0.55	0.84	-0.03
WDVI	0.98	0.97	0.73	0.92	0.94

**Figure 4:**
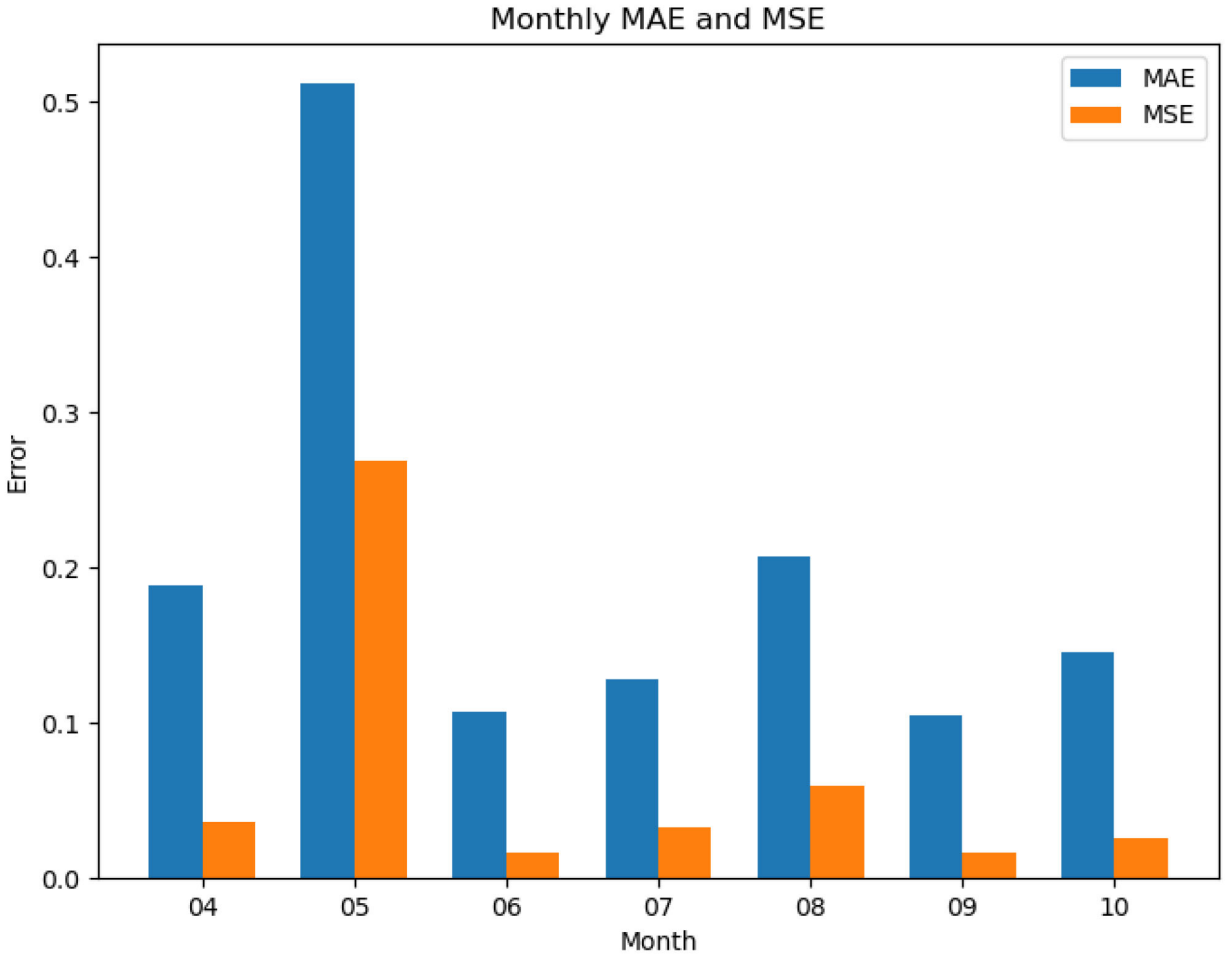
Monthly MAE and MSE of CNN model predictions for FPAR from April to October.

The prediction results of the CNN regression model for FPAR are presented in **Table 5**. The FPAR simulated by the model matches very well with the test set data. It is worth noting that the MAE and MSE were higher in July compared to other months (**Figure 4**). This is mainly due to the localized cloud cover in the Sentinel-2 imagery in July, which introduces noise into the vegetation index calculation and reduces the prediction accuracy. Overfitting was ruled out as the difference in MSE between the training and test sets was less than 5%, while the addition of Dropout and L2 regularization only slightly reduced the MAE by 2% in July, confirming that the elevated error was mainly due to data noise. Overall, the CNN model demonstrated significant reliability and superiority in FPAR estimation.

**Table 5:** CNN model training results for different months.

Month	R^2^	RMSE	EVS	MAE
April	0.84	0.0009	0.84	0.0057
May	0.95	0.0003	0.98	0.0137
June	0.98	0.0003	0.99	0.0127
July	0.74	0.0092	0.90	0.0830
August	0.98	0.0009	0.98	0.0229
September	0.99	0.0001	0.99	0.0058
October	0.90	0.0020	0.94	0.0315

**Figure 5:**
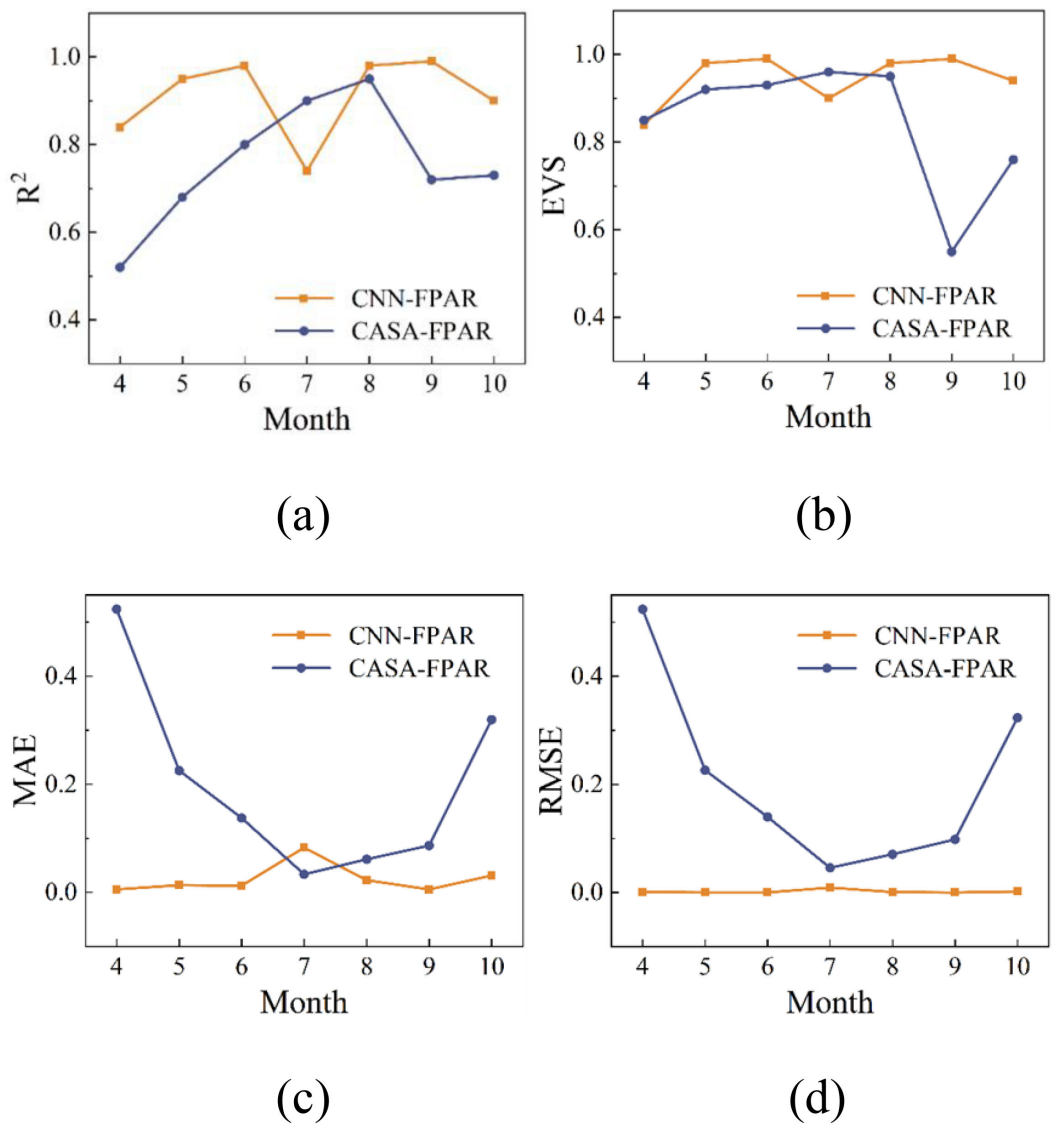
Comparison of original FPAR calculation methods and CNN model results: **(a)** R^2^, **(b)** Explained Variation Score, **(c)** Mean Absolute Error, **(d)** Root Mean Squared Error.

Analysis of the best feature combinations identified by the Recursive Feature Elimination (RFE) algorithm shows that DVI, GEMI, NDI45 and RVI are consistently included in the best set of features, suggesting that their importance extends beyond simple correlation ranking. To further validate these results, this study compares the CNN regression model’s FPAR predictions with those from the original CASA model. In the original CASA model, FPAR is estimated using the statistical relationships between NDVI and RVI. We compare FPAR results obtained from these statistical relationships with those calculated using radiative transfer models, and compute four evaluation indices, as shown in **Table 6**. According to the analysis in **Table 5**, the CNN model yields an average RMSE of 0.0020 and an average MAE of 0.0250, whereas the original CASA model shows an average RMSE of 0.2040 and an average MAE of 0.1984. The comparison of FPAR results between the CNN model and the original CASA model is illustrated in **Figure 5**. Except for a few months, the CNN model demonstrates a significant reduction in both RMSE and MAE. Specifically, the MAE for July from the CNN model is higher than that of the original CASA model, which may be due to spectral information errors caused by cloud cover. These results indicate that the CNN model developed in this study provides high accuracy and reliability in FPAR predictions (Ju and Roy, 2008; Zhu et al., 2010).

**Table 6:** Comparison of original FPAR estimates and actual FPAR for different months.

Month	R^2^	RMSE	EVS	MAE
April	0.52	0.5239	0.85	0.5237
May	0.68	0.2262	0.92	0.2255
June	0.80	0.1404	0.93	0.1378
July	0.90	0.0458	0.96	0.0338
August	0.95	0.0709	0.95	0.0615
September	0.72	0.0982	0.55	0.0869
October	0.73	0.3233	0.76	0.3198

### 3.2 FPAR NPP estimation results based on the improved CASA model

This study gathered 12 sets of actual NPP data with estimates from the improved CASA model, the original CASA model, the Geographic Remote Sensing Ecological Network (http://www.gisrs.cn), and the model enhanced by Professor Wenquan Zhu (Zhu et al., 2007). As shown in **Figure 5**, **Table 7**, and the accompanying analysis, the improved CASA model has a MAPE of 20.31%, which is lower than the original CASA model’s 28.92%, and significantly better than the 68% and 70.55% of the other two sources. Therefore, the MAPE of the improved CASA model is 8.61% lower than that of the original model, which indicates that the estimation error is smaller and the precision and reliability are higher. The higher error observed in the geo-remote sensing ecological network dataset and Zhu augmented model may be due to the fact that these data sources are less suitable for estimating crop NPP in the study area.

**Table 7:** Comparison of NPP estimation accuracy (MAPE) of different models.

Model	MAPE (%)
Improved CASA model	20.31
Original CASA model	28.92
Geographic RS Ecological Network	70.55
Zhu’s enhanced CASA model	68.00

**Figure 6:**
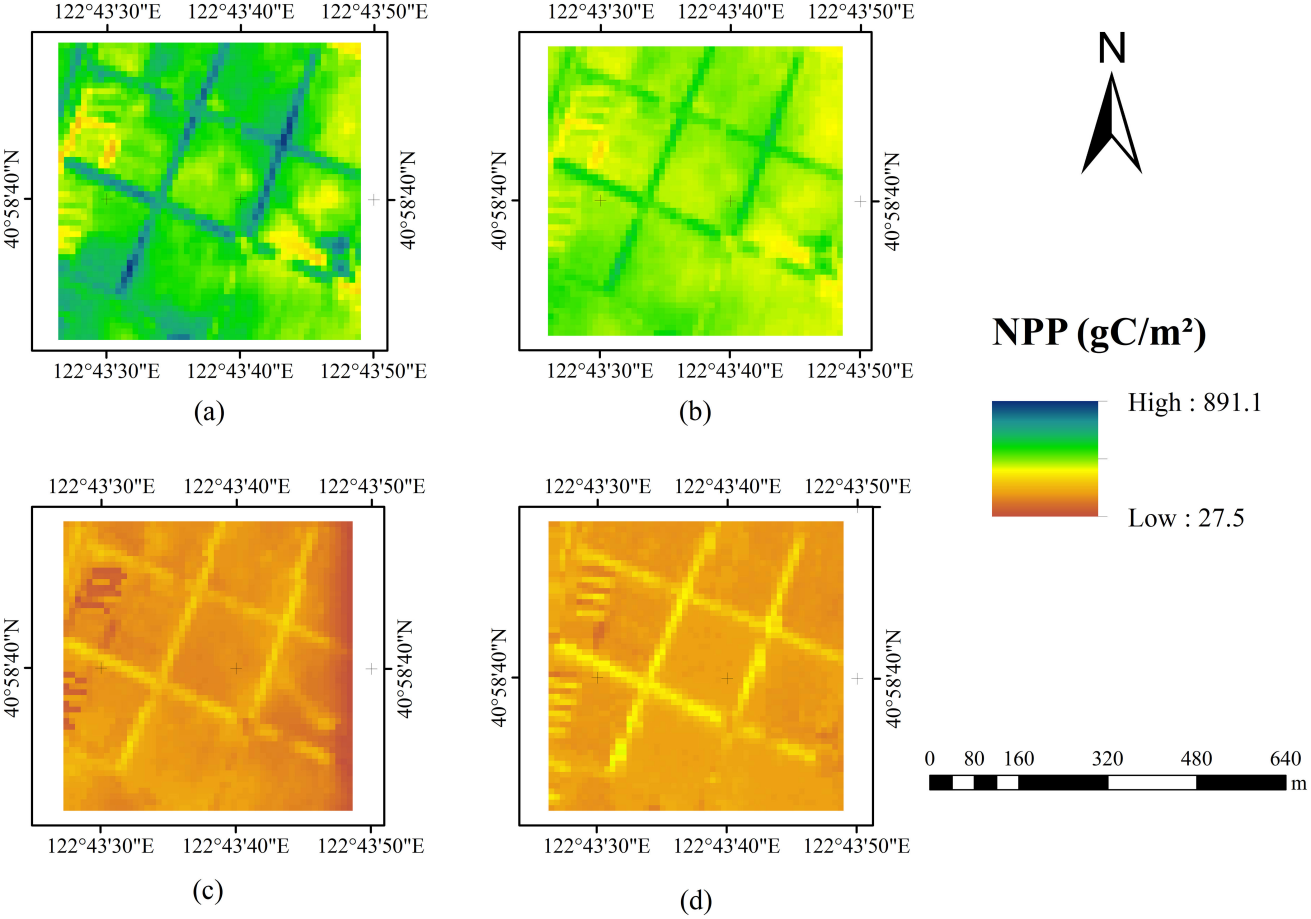
**(a)** Improved CASA model NPP result, **(b)** CASA model NPP result, **(c)** Zhu NPP model NPP result, **(d)** Geographic remote sensing ecological network NPP result.

Using the improved CASA model for the study area produced the NPP results displayed in **Figure 6** (a). The distribution of NPP values within the study area is broad, ranging from a minimum of 237.2 gC/m²/year to a maximum of 891.1 gC/m²/year, with an average of 535.3 gC/m²/year. Higher NPP values are generally found in the wooded areas adjacent to farmland, ranging from 680 to 890 gC/m²/year, while crop NPP ranges from 360 to 680 gC/m²/year. This difference is mainly attributed to the well-developed root systems of trees and their ability to store large amounts of carbon in their trunks, branches, and roots, whereas crop biomass is mainly concentrated in the harvested parts. These characteristics enable forests to accumulate more organic matter each year, resulting in higher NPP values (Myneni et al., 2001).

### 3.3 Analysis of NPP results for different crops

In this study, carbon content data for corn and rice samples were obtained through field NPP experiments. The average carbon content of corn samples was 46.09%, while that of rice samples was 41.37%. These data provide a solid foundation for calculating and analyzing vegetation NPP.

Comparisons between the measured NPP results and those estimated by the improved model revealed that the average absolute error for corn NPP estimates decreased by 29% with the improved model. This substantial reduction in error indicates that the revised model more accurately captures the growth characteristics and productivity changes of corn. For rice, the average error was reduced by 5.79% with the improved model. Although this improvement is relatively modest, it still demonstrates the model’s adaptability and effectiveness in handling different crop types.

In order to improve the regression accuracy of FPAR estimation in general, this study developed a CNN-based model and compared its performance with that of Snot Boosted Decision Tree (GBDT) and Extreme Gradient Boosting (XGBoost). These models were evaluated by metrics such as R^2^, mean square error (MSE), explained variance score (EVS) and mean absolute error (MAE). The comparison results are summarized in **Table 8** and show that the CNN model achieves the highest accuracy (R^2^ = 0.98) and the least prediction error (MSE = 0.0003, MAE = 0.0127), outperforming GBDT and XGBoost.

**Table 8:** Comparison of the CNN model with GBDT and XGBoost models.

Model	R^2^	MSE	EVS	MAE
CNN	0.98	0.0003	0.98	0.0127
GBDT	0.85	0.0025	0.87	0.0528
XGBoost	0.89	0.0016	0.85	0.0432

Further analysis of the NPP data for corn and rice showed that in the study area, rice NPP ranged from 376.3 to 644.1 gC/m²/year, with an average of 503.9 gC/m²/year. In contrast, corn NPP ranged from 413.3 to 668.5 gC/m²/year, with an average of 526.0 gC/m²/year. However, based on the measured NPP results, the average NPP for corn was 723.8 gC/m²/year, and for rice, it was 577.1 gC/m²/year. This discrepancy may be attributed to corn’s higher photosynthetic efficiency, leading to greater carbon content and, consequently, higher NPP for corn compared to rice (Myneni et al., 2001; Ni, 2004; Wang et al., 2010).

## 4 Discussion

FPAR is strongly correlated with vegetation biomass, health and environmental conditions. Among the vegetation indices, SAVI, MSAVI and TSAVI showed strong and stable correlations with FPAR due to their ability to reduce soil background effects (Richardson and Wiegand, 1977; Tucker, 1979; Huete et al., 1997; Delegido et al., 2011). Furthermore, the feature selection results indicate that indicators such as DVI, GEMI, NDI45, and RVI are consistently included in the optimal feature set, highlighting their critical role in improving the accuracy of FPAR estimation. Specifically, DVI quantifies vegetation growth by calculating the difference between the near-infrared (NIR) band and the red light band, making it an important indicator for assessing vegetation biomass and distinguishing vegetation types (Gunathilaka, 2021). GEMI enhances the accuracy of vegetation information extraction by minimizing soil and atmospheric effects (Pinty and Verstraete, 1992). NDI45, based on the mid-infrared (MIR) and red light bands, better reflects vegetation moisture content and health status (Khan et al., 2020). RVI, as the ratio of the near-infrared and red light bands, can effectively identify areas with high vegetation coverage (Gupta, 1993). These results indicate that combining multiple indices can enhance the model’s adaptability to different crop growth conditions.

This study mainly focused on corn and rice, which limits the generalizability of the results. Different crop species, such as wheat or soybean, may exhibit distinct photosynthetic pathways and growth characteristics, which could influence the accuracy of NPP estimation. Future research will extend the model to more crop species to enhance its applicability across diverse agricultural systems.

In summary, FPAR is closely related to vegetation biomass, health status, and environmental factors.

**Figure 7:**
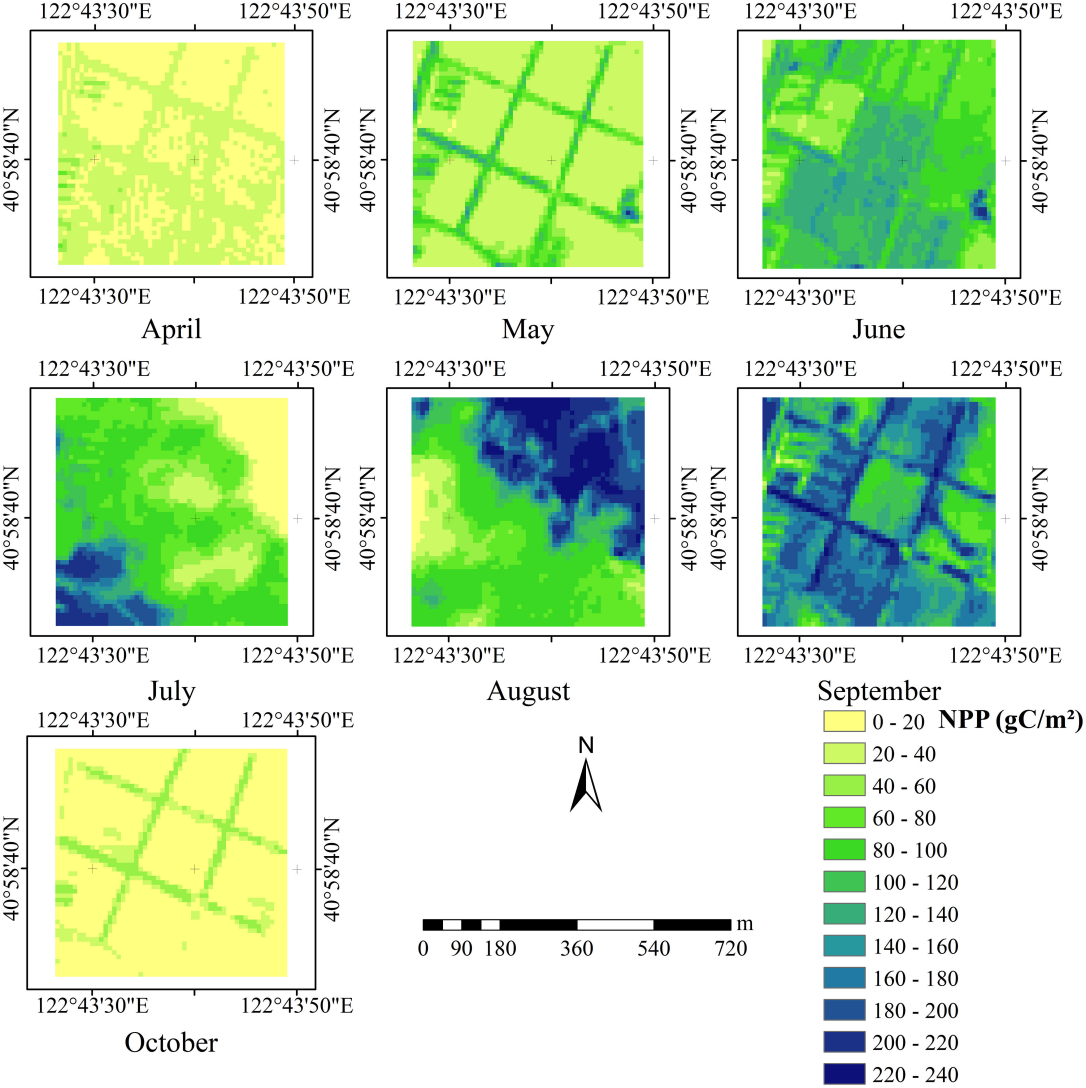
Distribution of NPP from April to October in the study area.

Crop growth relies on an optimal environment, where temperature, moisture, sunlight, and soil nutrients are critical factors. These elements influence the rates of photosynthesis and plant respiration, ultimately regulating NPP (Hatfield et al., 2011; Lobell and Gourdji, 2012). This study analyzes the monthly cumulative NPP results to observe variations in crop NPP across different growth stages. As shown in **Figure 7** rops exhibit the highest NPP in June, July, August, and September. It displays the monthly NPP simulation results derived from the original CASA model using empirical FPAR estimates. This peak is likely associated with favorable temperatures, adequate rainfall, and high solar radiation during these months, which jointly promote biomass accumulation by enhancing chlorophyll synthesis, stomatal conductance, and light use efficiency (Xu et al., 2021).

In the study area, summer begins in June, a season typically conducive to crop growth due to elevated temperatures, ample rainfall, and extended daylight hours. These meteorological conditions enhance photosynthetic efficiency, support nutrient uptake, and provide sufficient energy for biomass accumulation (Lobell and Asner, 2003; Hatfield and Prueger, 2015). June marks the start of the rapid growth phase, during which crops reach their maximum leaf area and highest photosynthesis rates, resulting in peak NPP. Although some crops may enter the senescence phase by September, overall photosynthetic activity and organic matter production remain high during this period.

Different crop species respond variably to temperature, rainfall, and sunlight. For instance, corn and wheat demonstrate higher photosynthetic efficiency under elevated temperatures, while rice requires ample water. The high NPP observed during these months corresponds with the growth characteristics of these crops.

In summary, temperature, rainfall, and solar radiation are significant environmental factors affecting crop NPP. Particularly during June, July, August, and September, these factors contribute to peak photosynthetic efficiency and monthly NPP. High temperatures accelerated enzymatic activities associated with photosynthesis, while adequate rainfall ensured the availability of water for nutrient transport and biomass accumulation. At the same time, increased solar radiation provided ample energy inputs, further increasing productivity. These findings provide a scientific basis for optimizing crop planting and management, potentially enhancing crop yield and quality.

Future research could further investigate the effects of other environmental variables such as soil nutrients, topography, and pest and disease pressure, which also affect carbon assimilation and plant health. In addition, the integration of advanced modelling techniques such as deep learning (e.g. CNN) can further improve the accuracy and scalability of net productivity estimates by capturing non-linear interactions between multiple sources of remote sensing inputs. A more comprehensive understanding of these factors will help refine precision agriculture practices to support sustainable crop production in complex agroecosystems.

The CNN-based FPAR estimation method proposed in this study outperforms the original CASA model in terms of accuracy. This improvement is primarily attributed to the CNN model’s ability to capture complex nonlinear relationships and multi-scale features. The original CASA model relies on the statistical relationships between FPAR and vegetation indices such as NDVI and RVI, which present inherent limitations. In areas with high vegetation coverage, as biomass and canopy density increase, the absorption and reflectance of light by vegetation leaves reach a saturation point. Beyond this threshold, NDVI and RVI values no longer exhibit significant changes with increasing biomass, leading to an underestimation of FPAR when relying solely on these indices. In this study, the RFE algorithm was employed to select the optimal feature set, while the CNN model was utilized to capture the nonlinear response between vegetation indices and FPAR, significantly enhancing the accuracy of FPAR retrieval.

The improved CASA model demonstrates higher accuracy in NPP estimation, primarily due to the enhanced precision of FPAR retrieval. In the CASA framework, NPP estimation is closely linked to FPAR, as NPP is computed based on the principle that vegetation absorbs PAR and converts it into biomass. Since FPAR quantifies the proportion of PAR absorbed by vegetation, its estimation accuracy directly affects the precision of NPP calculations. With the improved FPAR retrieval accuracy, the enhanced CASA model can more accurately determine the actual amount of PAR absorbed by vegetation, thereby achieving more precise NPP estimations.

Although this study focuses on Haicheng City, the proposed model exhibits considerable potential for generalization. The core of the model lies in the integration of satellite remote sensing data and meteorological data, a widely adopted approach for studying NPP and the FPAR. This methodology is inherently applicable across various ecosystems. While the model has been calibrated and validated within the specific environmental conditions of Haicheng City, its applicability extends beyond this region.

To adapt the model for other regions, several key steps must be undertaken. First, high-quality satellite remote sensing data for the target area must be collected over a sufficiently long temporal span, alongside meteorological data—including temperature, precipitation, and solar radiation—corresponding to the same time period. Second, since vegetation types vary across regions, the model’s maximum light-use efficiency parameter should be adjusted accordingly. This adjustment should be based on existing research findings and tailored to the specific vegetation characteristics of the target area. Third, following the research framework established for Haicheng City, FPAR and vegetation index data from the target region should be gathered and input into the CNN model, with parameter tuning performed to optimize simulation accuracy. Finally, the predicted FPAR data, along with meteorological inputs, can be incorporated into the CASA model to derive the NPP values for the target region.

This study employed the CASA model within the light use efficiency framework to simulate crop NPP. Although significant progress has been made in improving NPP estimation accuracy, several uncertainty factors continue to affect the precision and reliability of the results.

**Figure 8:**
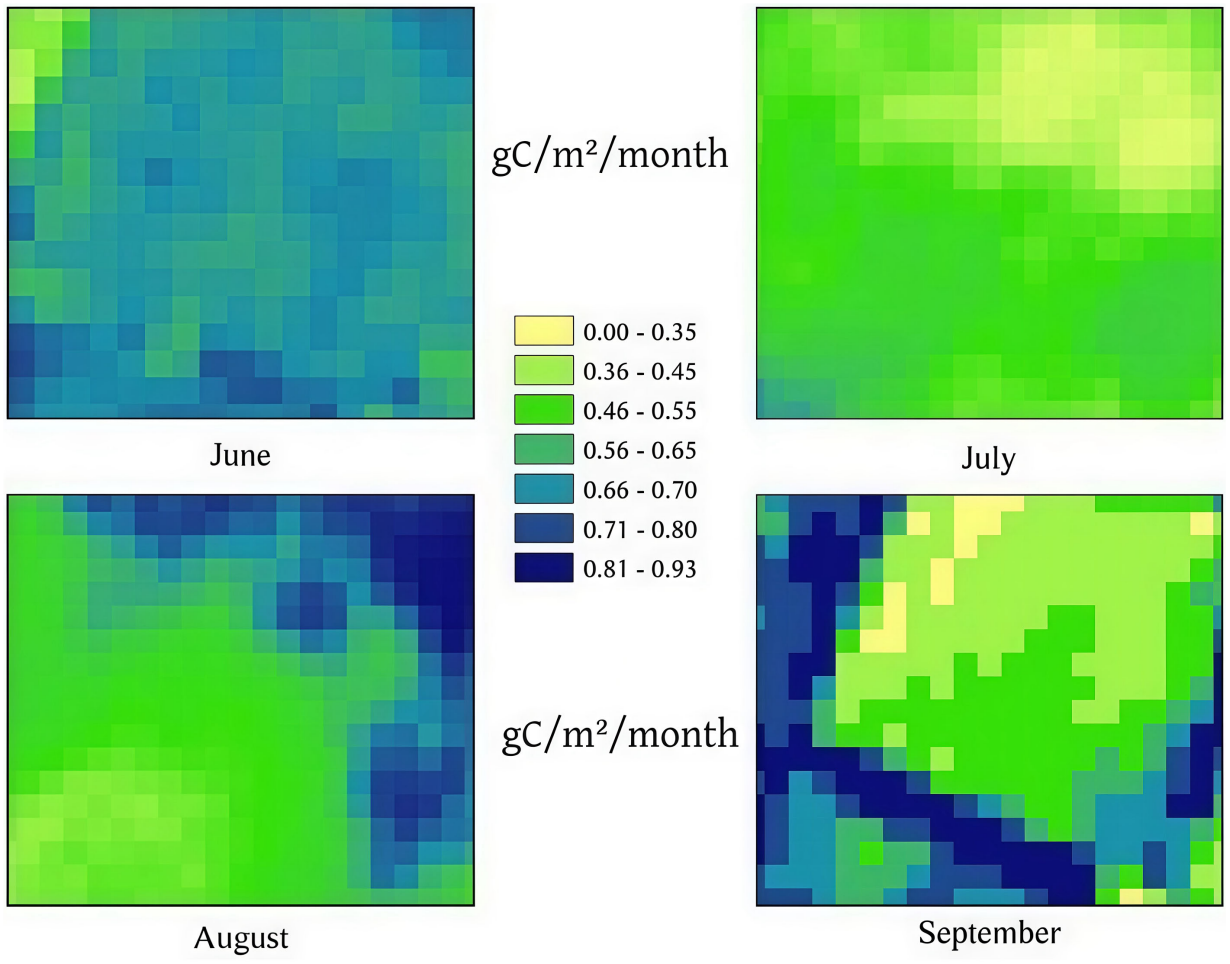
Spatial distribution of monthly NPP estimated by the CNN model for June, July, August, and September.

The spatial and temporal resolution of remote sensing data directly influences the accuracy of NPP estimation. High-resolution imagery from the Sentinel-2 satellite captures detailed vegetation spectral information, offering significant advantages. However, certain conditions, such as cloud cover and atmospheric variations, can lead to data loss or introduce errors in specific regions. While the high-resolution imagery provides detailed spectral information, it is also susceptible to potential error sources such as sensor noise and changes in illumination conditions. These errors may introduce noise into the vegetation indices used for FPAR estimation, thereby impacting the accuracy of NPP calculations. In this study, Sentinel-2 imagery of the study area from July and August exhibited small-scale cloud cover. Clouds obscure portions of the land surface, resulting in incomplete spectral data for these areas. For FPAR estimation, cloud cover impedes the accurate calculation of vegetation indices, which are critical for deriving FPAR. **Figure 8** compares FPAR values for the same region from June to September, highlighting cloud-affected areas in July and August. It presents the monthly NPP results obtained by inputting CNN-estimated FPAR into the CASA model, thereby incorporating improved FPAR accuracy. As shown in the figure, FPAR values in cloud-affected regions are likely underestimated. The inaccuracies in FPAR caused by cloud cover directly affect NPP estimation. Reduced FPAR values lead to a decrease in APAR, ultimately resulting in lower NPP estimates for cloud-covered regions. This underscores the necessity of addressing cloud-induced data gaps to improve the reliability of NPP calculations.

The current study was conducted within a relatively limited region in Haicheng City. Although the framework integrating CNN-derived FPAR with the CASA model shows promising results, its robustness should be further validated across larger and more heterogeneous agricultural landscapes. Future applications will focus on expanding the spatial scope to evaluate model transferability at regional and national scales.

Previous studies have improved CASA-based NPP estimation by optimizing light use efficiency parameters or integrating alternative vegetation indices (Ju and Roy, 2008; Yu et al., 2009). Our approach complements these efforts by introducing CNN-based FPAR retrieval, which captures nonlinear interactions among indices. This positions our work within the broader literature on CASA model enhancement and contributes to ongoing efforts to reduce uncertainties in large-scale NPP modeling.

Additionally, despite the incorporation of actual NPP data for corn and rice, the improved CASA model has certain limitations. Although optimizing the estimation method for FPAR has enhanced NPP accuracy, the estimation error for corn remains higher compared to rice. This discrepancy may be attributed to the uniform setting of the maximum light use efficiency parameter in the CASA model. In this study, a single maximum light use efficiency parameter of 0.389 was applied, which does not effectively differentiate between corn and rice. C4 plants (e.g., corn) exhibit higher photosynthetic efficiency than C3 plants (e.g., rice), as C4 plants utilize light energy more effectively and reduce respiration losses (Gowik and Westhoff, 2011). Consequently, the uniform parameter setting leads to estimation errors. To further improve NPP estimation accuracy, the CASA model’s maximum light use efficiency parameter needs to be optimized to account for the characteristics of different crops.

In summary, despite the significant advancements in refining the CASA model, future research should address and mitigate the aforementioned uncertainty factors to enhance the accuracy and reliability of NPP estimations.

Despite its demonstrated advantages, the CNN-based FPAR estimation framework also has certain limitations. First, it requires large amounts of high-quality training data, which may not always be available for all regions or crop types. Second, CNN training and inference are computationally intensive, potentially limiting large-scale or real-time applications. Future work could address these challenges through strategies such as transfer learning to reduce dependence on local training data, model compression to improve efficiency, or integrating CNN with process-based models to balance accuracy and interpretability.

## 5 Conclusions

This study demonstrates that combining deep learning with the CASA model can significantly improve the accuracy of estimating crop net primary productivity (NPP) under different environmental conditions. By integrating spectral reflectance (FPAR) estimated using convolutional neural networks (CNN), the improved model can more reliably represent spatial-temporal NPP patterns, providing support for theoretical research and practical applications in fields such as agricultural management, large-scale yield monitoring, and carbon cycle studies. The research results highlight the potential of combining advanced remote sensing technology with process-oriented models to enhance ecosystem productivity assessment. Future studies will further optimize model parameters, address data uncertainty issues (such as cloud contamination), and expand the application scope of this method to cover more crop types and geographical regions.

## Data Availability

The original contributions presented in the study are included in the article/supplementary material. Further inquiries can be directed to the corresponding author.
